# Supplementary material for: An oxygen-insensitive Hif-3α isoform inhibits Wnt signaling by destabilizing the nuclear β-catenin complex
Source: eLife. 2016 Jan 14;5:e08996. doi: 10.7554/eLife.08996 (PMC4769163; doi:10.7554/eLife.08996)
Supplement: Supplementary file 1. — DOI: http://dx.doi.org/10.7554/eLife.08996.027 [file elife-08996-supp1.docx]

**Supplementary file 1. Primers used in this study**

| Primer Name | Primer Sequence 5'-3' |
| --- | --- |
| F_Full-length Hif-3α2 | GAGATAAACAAGCAGCAGTC |
| R_Full-length Hif-3α2 | TGGACGCATGTGATGGACAG |
| F_Hif-3α2 expression (BamHI, pCS2-eGFP) | GCGGATCCGCCACCATGGAGATGGAAGG |
| R_Hif-3α2 expression (EcoRI, pCS2-eGFP) | TACCGGAATTCCAGTCAGGGATGGCAATGTTTCCGC |
| F_Hif-3α2 expression (BamHI, pCS2-Flag) | CGGGATCCGAGATGGAAGGAGTGGAGAAGTTCTTCGCCCTC |
| R_Hif-3α2 expression (EcoRI, pCS2-Flag) | CGGAATTCTCAAGTCAGGGATGGCAATGT |
| F_ΔTAD | GCGGATCCGCCACCATGACCTTCCTGCCCCAGAGTG |
| R_ΔTAD | TACCGGAATTCCAGTCAGGGATGGCAATGTTTCCGC |
| F_ΔLZ | GCGGATCCGCCACCATGGAGATGGAAGG |
| R_ΔLZ | CGGAATTCCCGGGTCCCTGTCGGTTAAAAG |
| F_A36G | TTGGACATGCTAGGTCCATATATCTCC |
| R_A36G | GGAGATATATGGACCTAGCATGTCCAA |
| F_P37A | GATTTGGACATGCTAGCTGCATATATCTCCATGGACG |
| R_P37A | CGTCCATGGAGATATATGCAGCTAGCATGTCCAAATC |
| F_P47S | CGATGACTTCCAGTCGACCTTCCTGCCCCAG |
| R_P47S | CTGGGGCAGGAAGGTCGACTGGAAGTCATCG |
| F_M1 | GGCTGTGGATGAGGCTGCTGCGGACATGCTAGCT |
| R_M1 | AGCTAGCATGTCCGCAGCAGCCTCATCCACAGCC |
| F_M2 | CTCCATGGACGATGCCGCCGCGTTGACCTTCCTGC |
| R_M2 | GCAGGAAGGTCAACGCGGCGGCATCGTCCATGGAG |
| F_Hif-3α2 GST (BamHI, pGEX-KG) | CGGGATCCGAGATGGAAGGAGTGGAG |
| R_Hif-3α2 GST (HindIII, pGEX-KG) | CCTAAGCTTTCAAGTCAGGGATGGCAA |
| F_Hif-3α2 gRNA | GATCACTAATACGACTCACTATAGCCCCGCTGAAGAGCTGCCCAGTTTTAGAGCTAGAAAT |
| R_Hif-3α2 gRNA | AAAAGCACCGACTCGGTGCC |
| F_Hif-3α2 gRNA F1_test | CAAAGTCCCAACAGTGTCCC |
| R_Hif-3α2 gRNA R1_test | GGTTAGCATTTCCAAAGGTG |
| F_Hif-3α2 gRNA F2_test | CGCAAGCTTCTGTCTCCAATA |
| R_Hif-3α2 gRNA R2_test | TGACTGTCCTTTCGGGGTTA |
| R_Hif-3α2 gRNA R3_test | CTTGAGGGCGAAGAACTTCT |
| F_genotyping-F2 | CGCAAGCTTCTGTCTCCAATA |
| R_genotyping-R2 | TGACTGTCCTTTCGGGGTTA |
| R_genotyping-R3 | CTTGAGGGCGAAGAACTTCT |
| F_genotyping-RT-F1 | CATGGAGATGGAAGGAGTGG |
| F_genotyping-RT-F2 | CAGCGAGCCCCGCTGAAGAGC |
| R_genotyping-RT-R | CTTCCAAACACCGTTTCCTAGA |
| F_β-actin_qRT-PCR | ACAGGGAAAAGATGACACAG |
| R_β-actin_qRT-PCR | AGAGTCCATCACGATACCAG |
| F_her4_qRT-PCR | AGGAGAACTGAACACAAGACAC |
| R_her4_qRT-PCR | TGCTGTTGATTCGCTCTCG |
| F_foxj1a_qRT-PCR | TACTTCCGCCACGCAGAT |
| R_foxj1a_qRT-PCR | TTGCCCGGTTCATCCTTCTG |
| F_cacna1c_qRT-PCR | TTGCAAACTGTGTGGCCTTAGCTG |
| R_cacna1c_qRT-PCR | TTCCGCAGGTATGCATTAGGGTGA |
| F_p53_qRT-PCR | CAGTCTGGCACAGCAAAATC |
| R_p53_qRT-PCR | TTTGCCAGCTGACAGAAGAG |
| F_gapdh_qRT-PCR | ACTGGAGAGCTCAAGTGCAG |
| R_gapdh_qRT-PCR | GTCCACGTCTCTCAAGCCAA |
| F_gli1_qRT-PCR | ACGCAGGATCCACTTCTTGG |
| R_gli1_qRT-PCR | CTCCTGCGTGTCGAACTCTT |
| F_dusp6_qRT-PCR | GCACAAGAGACTTTGGCGTG |
| R_dusp6_qRT-PCR | TCGATCTGCACGGGTTTGAA |
| F_boz_qRT-PCR | GATGTACTGCTGCTGCGTTCC |
| R_boz_qRT-PCR | CTGCTCCGTCTGGTTGTCG |
| F_chd_qRT-PCR | ACGCCTGCTGCCATACAAT |
| R_chd_qRT-PCR | CACTGAGGGTCCACCGAGA |
| F_vox_qRT-PCR | GCGCGCGGATTTTCTGCTGC |
| R_vox_qRT-PCR | GGGAACGGGAGCCGCTGTCT |
| F_vent_qRT-PCR | GGAGAGTGATGACAGTGAAGTAGA |
| R_vent_qRT-PCR | ACAGCGGGATAGAGGAAGT |
| F_sox17_qRT-PCR | CCGCTCTCAGACTCCAAATC |
| R_sox17_qRT-PCR | TTACTCAGCTCCGCATTGTG |
| F_myod1_qRT-PCR | GAGGACGAGCACGTGAGGGC |
| R_myod1_qRT-PCR | TCATGGTGGCGGCTTTGCGA |
| F_myog_qRT-PCR | CCTTCAGACCAGCTTTCACTGA |
| R_myog_qRT-PCR | CCAAGGCTTGTCTAACTTGCAA |
